# Metastatic Gallbladder Melanoma Presenting as Acute Emphysematous Cholecystitis

**DOI:** 10.1155/2018/5726570

**Published:** 2018-05-08

**Authors:** Natalie Hall, Nicole L. Grenier, Samir A. Shah, Richard Gold, Edward Feller

**Affiliations:** ^1^Alpert Medical School of Brown University, Providence, RI, USA; ^2^Division of Dermatology, Alpert Medical School of Brown University, Providence, RI, USA; ^3^Division of Gastroenterology, Department of Medicine, Alpert Medical School of Brown University, Providence, RI, USA; ^4^Division of Diagnostic Imaging, Alpert Medical School of Brown University, Providence, RI, USA; ^5^Division of Medical Education, Alpert Medical School of Brown University, Providence, RI, USA

## Abstract

Malignant melanoma is an aggressive tumor with a high potential for distant metastases, including spread to the gallbladder where it represents more than half of all metastases detected at autopsy. Yet, it is rarely symptomatic in life and is a rare cause of acute cholecystitis. Emphysematous cholecystitis is a rare, potentially fatal variant of acute cholecystitis characterized by the presence of gas in the gallbladder lumen or wall. We report a 77-year-old woman with acute emphysematous cholecystitis as the initial feature of recurrent melanoma metastatic to the gallbladder. This exceptional association highlights the need to consider a relapse of malignancy when assessing unexplained abdominal symptoms in any patient with a prior history of melanoma.

## 1. Introduction

Melanoma is the most common malignancy to metastasize to the gallbladder, accounting for as many as 50–65% of all gallbladder metastases found at autopsy [1, 2]. Yet, it is rarely symptomatic in life and is a rare cause of acute cholecystitis [3]. In more than 90% of cases, acute cholecystitis is due to a gallstone blocking the cystic duct; rarely the blockage is caused by a benign or malignant tumor or a hematoma [4, 5]. Emphysematous cholecystitis is a rare, potentially fatal variant of acute cholecystitis characterized by the presence of gas in the gallbladder lumen or wall. Emphysematous cholecystitis may be due to gallbladder wall ischemia and subsequent necrosis with invasion of gas-producing bacteria within the gallbladder wall or lumen [6].

We report a patient with acute emphysematous cholecystitis as the presenting feature of a recurrent melanoma metastatic to the gallbladder. This exceptional association underscores the need to consider relapse of malignancy when evaluating unexplained abdominal complaints in any patient with a known history of melanoma.

## 2. Case Report

A 77-year-old female presented with one day of sharp, severe right upper quadrant pain and nausea and vomiting. The pain did not radiate. Two years previously, she had undergone wide local excision of a superficial spreading melanoma of the upper back with a thickness of > 2.0 mm (Stage IIa, T_3a_ N_0_ M_0_). Sentinel lymph node biopsy was negative for tumor. Her physicians deemed her to be in complete remission.

On physical exam, she was afebrile and anicteric. Vital signs were within normal limits without postural change. Abdominal exam was notable for marked right upper quadrant tenderness with guarding, but without signs of peritoneal irritation, mass, or organomegaly. White blood cell count was elevated to 13.1 × 10^3^/*μ*L, hemoglobin was within normal limits, and creatinine  was  2.6 mg/dL. Serum liver chemistries, amylase, and lipase were all normal.

### 2.1. Hospital Course

**Figure 1:**
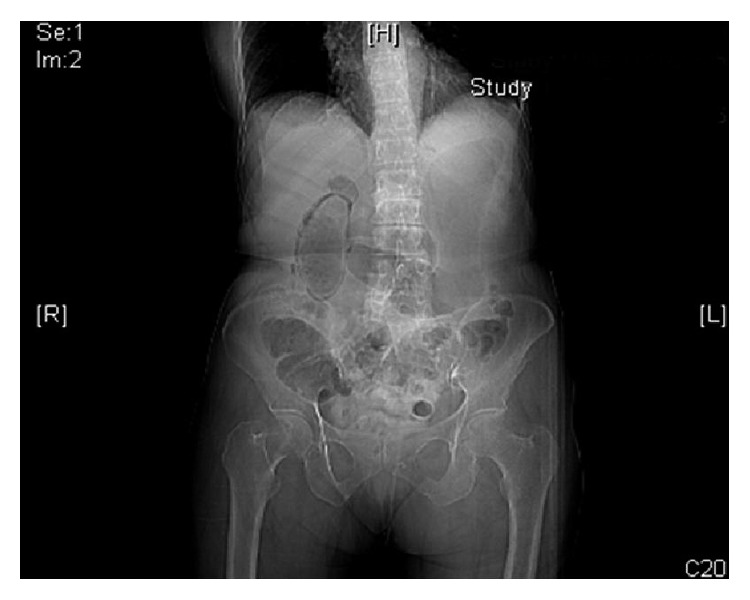
Plain abdominal radiograph. Air outlining the gallbladder.

**Figure 2:**
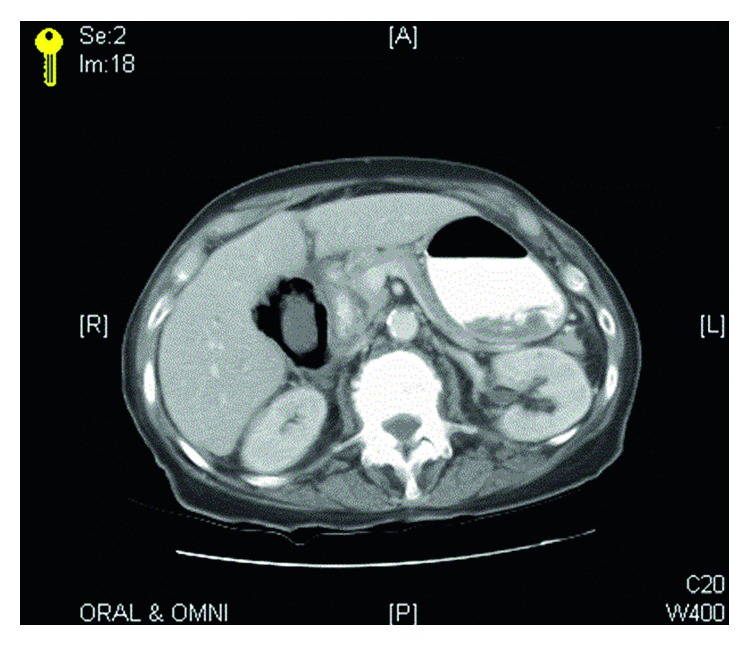
CT scan. Extensive intramural gas with a thickened gallbladder wall surrounded by marked inflammatory changes.

She received imaging studies in the emergency department. Plain abdominal flat plate revealed air outlining the gallbladder (Figure 1). Helical CT images through the abdomen after oral and intravenous contrast showed extensive intramural gas with a thickened gallbladder wall surrounded by marked inflammatory changes. Imaging was consistent with a perforated, emphysematous gallbladder (Figure 2).

The patient was admitted and started on intravenous ampicillin-sulfabactam. Urgent open cholecystectomy was performed after an unsuccessful attempt at laparoscopic cholecystectomy. The surgical specimen revealed perforated emphysematous cholecystitis and biliary sludge. A 1 cm brownish polypoid lesion projected into the lumen, arising from the mucosa of the fundus, but not obstructing the cystic duct. The lesion was positive for malignant melanoma by immune-histochemistry. No microorganism was isolated. Subsequent chest and abdominal CT scans revealed unsuspected, irregular lesions in the left lung consistent with metastatic melanoma, a probable metastatic lesion in the liver, and enlarged lymph nodes in the upper abdomen. After discussion, the patient decided to delay treatment until she became symptomatic. She died within months.

## 3. Discussion

Malignant melanoma represents less than 1% of all cancers in the United States. Metastases to the gastrointestinal (GI) tract are uncommon. Gallbladder involvement is found postmortem in up to 15% of patients dying of metastatic melanoma [1, 3]. Symptomatic melanoma of the gallbladder is rare; published reports consist of a few dozen, isolated case reports or small case series. There are no large case series or reviews of collected experience to be able to compare the number of asymptomatic to symptomatic cases. Dissemination of malignant cells to distant sites occurs most commonly via regional lymph nodes [7]. Hematogenous spread is less frequent. The gallbladder mucosa may also be involved through the implanting of tumor cells carried in bile [2].

The pathophysiology of EC is thought to be due to vascular ischemia leading to tissue necrosis. Inadequate blood supply to the gallbladder is commonly secondary to other disorders such as atherosclerosis, arterial embolism, vasculitis, or systemic hypotension or hypoperfusion. Ischemic injury can facilitate bacterial invasion with subsequent accumulation within the gallbladder wall [8].

The interval between the diagnosis of a primary cutaneous melanoma and a metastatic relapse may be prolonged; rarely, decades intervene before a symptomatic recurrence. Thus, delay in diagnosis is common when the index of suspicion is low so many years after treatment of a primary cutaneous melanoma. Delayed diagnosis may also occur when metastases are the presenting feature of a previously undiagnosed cutaneous malignancy. Further, there have been reports of primary mucosal melanoma of the oral cavity, uveal tract, meninges, and vaginal and anorectal region metastasizing to the gallbladder without a history of cutaneous melanoma [9, 10]. In 4–10% of patients with metastatic melanoma, the primary tumor is never identified, possibly as a result of spontaneous regression [11]. Careful inspection of the skin and other potential origins including mucosal sites such as eyes, oral cavity, anus, urethra, and vagina is vital since primary mucosal melanoma has no cutaneous source [10].

When symptomatic, gallbladder metastases most commonly present as abdominal pain suggestive of acute cholecystitis due to a gallstone migrating into the cystic duct. Acute emphysematous cholecystitis at presentation may also mimic uncomplicated acute cholecystitis [8, 12]. The dangerous emphysematous form can also present with nonspecific chronic or subacute upper abdominal pain, jaundice due to bile duct obstruction or the hemolytic effect of clostridia species [13, 14], the unexpected imaging finding of air in the gallbladder wall, a polypoid lesion or biliary fistula on imaging [15], or GI bleeding from an ischemic or necrotic gallbladder source, termed “hemobilia” [16, 17]. Other uncommon presentations include hypotension, isolated fever, or sepsis. Our patient represents an exceptional case of melanoma metastasizing to the gallbladder presenting as emphysematous cholecystitis.

Diverse abdominal disorders can cause gas or air accumulation in the biliary tree, termed “pneumobilia.” Causes include incompetent sphincter of Oddi, biliary enteric surgical anastomosis, biliary enteric fistula, acute cholangitis, cholangiography from ERCP or PTC, gallbladder infarction, hepatic abscess, or abdominal trauma [18].

As many as 50% of cases of emphysematous cholecystitis are acalculous [6]. On macroscopic exam, our patient had biliary sludge composed of particulate matter and bile and it occurs when solutes precipitate in bile. Cystic duct obstruction was absent. The inciting event in the emphysematous variant is believed to be vascular ischemia leading to tissue necrosis in which gas-producing bacteria may proliferate. Typical acute calculous cholecystitis may also progress, less commonly, to gallbladder wall ischemia with necrosis and resulting invasion of gas-producing bacteria within the wall [3]. Abdominal wall crepitus overlying the gall bladder may rarely be detected, although absent in our patient. No organism was isolated in our patient, which may have been due to a laboratory error or other technical problem in identification.

As many as 50% of patients with emphysematous cholecystitis have diabetes mellitus, advanced age, or peripheral vascular disease, all of which increase the risk of ischemic damage to organs [6]. Other risk factors include male gender, immunocompromised state, recurrent gallstones and cholecystitis, alcoholism, and uremia. The responsible gas-forming bacteria are usually anaerobes, most commonly *Clostridium* species as well as *Escherichia coli*, *Klebsiella* spp, *Proteus* species, *Aerobacter aerogenes*, *Staphylococcus*, anaerobic *Streptococcus*, and *Salmonella derby* [6]. Despite gas in the gallbladder wall, no pathogen was isolated from our patient. In a 2016 study, Ambe et al. reported more than 12,000 patients with emphysematous cholecystitis compared to a control group of 30,000 with uncomplicated acute cholecystitis. The mortality rate was 2.8% from EC and 1.2% from AC [6]. Complications of emphysematous cholecystitis may be life threatening, such as gallbladder gangrene or rupture [19], GI bleeding, hypotension, shock, bacteremia or sepsis, and pericholecystic or visceral abscess [20]. Gas may also disseminate to subcutaneous tissue, the peritoneal and retroperitoneal cavity [14], mediastinum [21], and as far as the lower extremities, causing metastatic gas gangrene of the leg [22].

The imaging appearance of metastatic melanoma is variable, including multiple flat mural nodules, infiltrative lesions, or a single polypoid lesion [1]. Associated focal gallbladder wall thickening is common. These lesions can be indistinguishable on ultrasound and CT imaging from benign gallbladder polyps. Due to the presence of melanin, these tumors demonstrate T1 hyperintensity with variable T2 hypointensity depending on melanin concentration and the presence of necrosis and/or hemorrhage. Melanoma metastases demonstrate robust enhancement on postcontrast CT and MR [23].

Abdominal CT scan is the prime imaging modality to diagnose emphysematous cholecystitis. In emphysematous cholecystitis, gas can obscure gallbladder visualization on ultrasonography. Misdiagnosis occurs because the gallbladder erroneously appears collapsed and lacking wall thickening [24]. In our patient, a plain abdominal radiograph was the important initial imaging clue, demonstrating air outlining the gallbladder (Figure 1). CT is also useful for visualizing fulminant sequelae such as gangrene, perforation, and pericholecystic abscess.

Polypoid gallbladder lesions include any lesions arising from the gallbladder mucosa with a prevalence as high as 3–7% on ultrasound [23]. Benign cholesterol polyps account for the majority of lesions. Primary gallbladder carcinoma is the most frequent malignant polypoid lesion. Melanoma is the most common metastatic gallbladder tumor. Metastatic gallbladder lesions have been reported from a variety of other sites, including lung, kidney, lymphoma, or via direct invasion from a contiguous hepatocellular carcinoma or cholangiocarcinoma [24, 25]. Imaging features of neoplastic masses may be difficult to distinguish from sludge, hematoma, mucosal folds, adherent cholesterol polyps, adenomyomatosis, or inflammatory polyps [24]. Endoscopic ultrasound (EUS) may be superior to transabdominal US for detection and diagnosis of gallbladder lesions because it provides better resolution of small lesions. EUS may also be useful in differentiation of benign from malignant features of a polyp [26].

## 4. Conclusion

Metastatic melanoma to the gallbladder is usually asymptomatic. Symptoms during life are rare and may mimic diverse, common, nonmalignant abdominal disorders. Our patient developed the exceptional variant of acute emphysematous cholecystitis. She had a known history of melanoma of the back, treated 2 years previously, and was thought to be in remission when abdominal pain occurred. This rare association emphasizes the need to consider recurrent malignancy when evaluating the gamut of unexplained abdominal complaints or uncertain imaging findings in patients with a known history of melanoma, even if diagnosed decades previously.

## Data Availability

Data (patient record) are available on request.
